# Differential expression of the heat shock protein Hsp70 in natural populations of the tilapia, *Sarotherodon melanotheron*, acclimatised to a range of environmental salinities

**DOI:** 10.1186/1472-6785-10-11

**Published:** 2010-04-29

**Authors:** Mbaye Tine, François Bonhomme, David J McKenzie, Jean-Dominique Durand

**Affiliations:** 1 Biologie Intégrative ISEM CNRS-UMR 5554 (Université Montpellier II), Station Méditerranéenne de l'Environnement Littoral, 1 quai de la Daurade, Sète 34200, France; 2 Institut de Recherche pour le Développement (IRD), UMR 5119 ECOLAG, campus IRD/ISRA de Bel Air, route des hydrocarbures, BP 1386, CP 18524 Dakar, Sénégal; 3 Max Planck Institute for Molecular Genetics, Ihnestrasse 63-73, D-14195 Berlin, Germany

## Abstract

**Background:**

The relationship between environmental variation and induction of heat shock proteins *(Hsps) *has been much documented under experimental conditions. However, very little is known about such induction in natural populations acclimatised to prevailing environmental conditions. Furthermore, while induction of stress proteins has been well documented in response to environmental contaminants and thermal stressors, little is known about whether factors, such as extreme salinity, are also potential inductors. The black-chinned tilapia *Sarotherodon melanotheron *is unusual for its ability to colonise estuarine environments in West Africa that are characterised by extremely high salinities. The relationships between mRNA levels of the 70 kDa heat shock protein (*Hsp70*) and Na^+^, K^+^-ATPase1α (*Naka*) in the gills, environmental salinity, and a life-history trait (condition factor) were investigated in wild populations of this species sampled from three locations in the Saloum estuary, at salinities ranging from 40 to 100 psu.

**Results:**

The highest *Hsp70 *and *Naka *mRNA levels, and the poorest condition factors were recorded in the most saline sampling site (100 psu). The *Hsp70 *and *Naka *mRNA were correlated amongst themselves and showed a direct positive correlation with environmental salinity, and a negative correlation with fish condition factor. Thus, the *Hsp70 *is constitutively overexpressed by *S. melanotheron *acclimatised to extreme hypersalinity.

**Conclusions:**

These results indicate that, although *S. melanotheron *can colonise extremely saline environments, the overexpression of *Hsp70 *combined with the higher *Naka *mRNA expression reveals that this represents a chronic stress. The induction of *Hsp70 *was, therefore, a biomarker of chronic hyper-osmotic stress which presumably can be linked to the impaired growth performance and precocious reproduction that have been demonstrated in the populations at the extremely saline sites.

## Background

The molecular family of heat shock proteins (*Hsps*) has been intensively studied in model organisms such as *Xenopus *and *Drosophila *submitted to stress in the laboratory [1-3] and, consequently, the physiological role of these proteins is becoming well understood at molecular and cellular levels in these models. The *Hsps*, in particular the 70 kDa (*Hsp70*) family, are constitutively expressed in cells under normal (non stressful) conditions and function as molecular chaperones, to keep other proteins from forming inappropriate aggregations. Aside from this function, *Hsps *are also implicated in the general protection of stressed cells and organisms [4,5]. Many studies have reported that exposure of organisms to such diverse stressors as temperature extremes, pollutants, anoxia, parasitism, predation, or competition; all elicit reversible increases in *Hsp70 *expression that serve to protect the organism against cellular damage [6-9]. The involvement of *Hsp70 *in the acclimation of fish to salinity changes has also been well documented experimentally [10,11]. In the silver sea bream, *Sparus sarba *[12], branchial expression of *Hsp70 *is increased in response to hypo- or hyper-osmotic shock.

It has been shown that increases in basal *Hsp70 *levels in stressful environments can be associated with reduced individual fitness [2]. This association of *Hsp *induction with fitness has been demonstrated for traits of development, growth and reproduction [13-15]. In *Drosophila melanogaster*, heat shock stress results in developmental defects and increased copy numbers of the gene encoding *Hsp70 *[16]. Higher muscle *Hsp70 *levels have been associated with lower growth rates in the Indo-Pacific sergean, *Abudefduf vaigiensis*, adapted to 32°C by comparison to those living at 28°C [17]. In the silver sea bream, *S. sarba*, the activity and mRNA levels of *Hsp70 *are lower around isoosmotic salinities, where the best growth performance is observed [12]. The *Hsp70 *may, therefore, provide a biomarker to identify stressful effects of environmental factors and to demonstrate a link between such factors and observed negative changes in life history traits of natural populations.

Another indicator of osmoregulatory challenges in fish is the sodium-potassium ATPase (Na+, K+-ATPase), a membrane protein which maintains ion gradients required for cell homeostasis and whose activity in the gills is related to either active ion secretion in hyper-osmotic conditions or active uptake in hypo-osmotic conditions [18-20]. Several *in vitro *studies have shown substantial correlations between environmental salinity and expression and/or activity of Na+, K+-ATPase (*Naka*) in teleost fish [21-26]. In the tilapia *Oreochromis mossambicus*, the activity of *Naka *has been used as a biochemical indicator of osmoregulatory stress [27]. Therefore, the induction of *Naka *can be used to assess the osmoregulatory status of fish in natural environments where salinity is the predominant abiotic stressor.

The black-chinned tilapia *S. melanotheron *has populations in the Saloum estuary in West Africa which experience salinities that range from brackish water to extremely hypersaline water (up to 130 psu), and where salinity can show considerable variations between wet and dry seasons [28]. Previous studies have shown that individuals inhabiting the highest salinities exhibit reduced growth rates [29,30] and precocious reproduction [30]. Although these phenotypic differences have been interpreted as indicative of hypersaline stress, this remains to be demonstrated.

In a previous study [31], multiple copies (singletons) of a gene encoding *Hsp70 *[ES882219] and *Naka *[ES881735] were isolated in a hypersaline SSH library created from gills of *S. melanotheron *acclimatised either to hypersaline water or to freshwater. Accordingly, Tine et al. [31] proposed that these genes must be indicators of the stressful effects of salinity on this species. The aim of this study was, therefore, to evaluate gill *Hsp70 *induction as a biomarker of constitutive organismal stress in natural populations of *S. melanotheron *sampled from environments with salinities ranging from 40 to 100 psu. Gill *Naka *expression levels were measured in parallel, as an indicator of the osmoregulatory status of the populations. The same sampling sites have previously been studied for the effects of salinity on life history traits and induction of osmoregulatory genes (growth hormone and prolactin) in *S. melanotheron *[29,30]. The relative expression of *Hsp70 *and *Naka *mRNA was quantified by real time PCR. The condition factor of the fish was also measured, as a proxy of physiological status [29,30].

## Results

### Branchial abundance of *Hsp70 *and *Naka *mRNA

**Figure 1:**
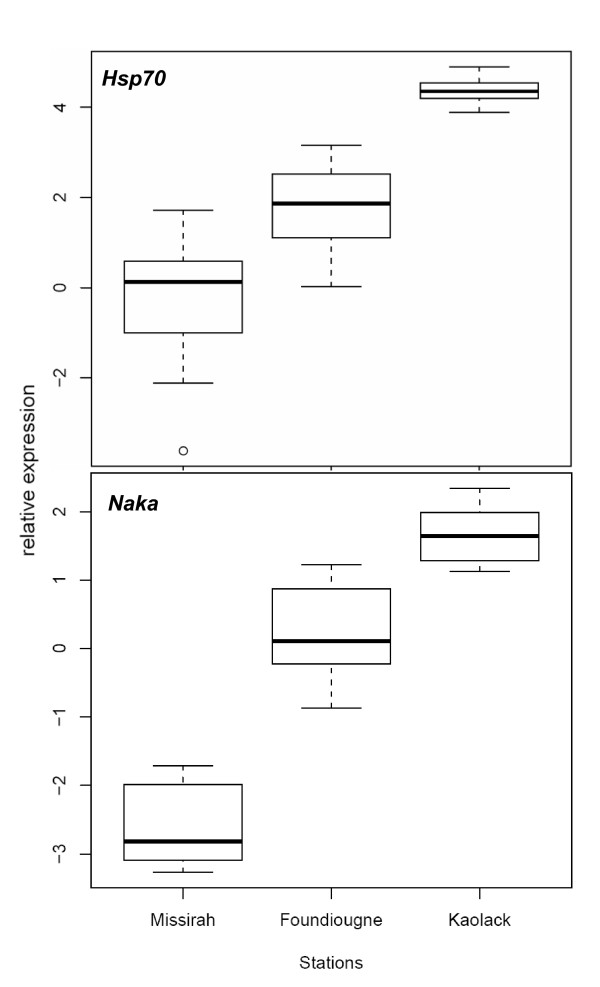
***Hsp70 *and *Naka *mRNA levels of the black-chinned tilapia *S. melanotheron *from three locations of the Saloum estuary**. Data are illustrated in box plots that contained the median (horizontal line) as well as the 25th and 75th percentiles (bottom and top edges of the boxes). The mRNA expression levels represent the relative expression normalized to β-actin and are expressed relative expression as log2-transformed data.

The rt-PCR analysis showed that the two primer pairs amplified a simple specific product with an efficiency of 1.95 for *Hsp70*, 1.91 for *Naka *and 1.89 for β-actin. We therefore calculated relative abundance to correct for the differences in efficiency. A significant impact of salinity on *Hsp70 *relative expression was observed within the Saloum estuary, fish sampled at the upstream location with highest salinity (Kaolack) had higher *Hsp70 *mRNA levels than fish sampled in less saline stations (Foundiougne and Missirah) (Figure 1). The amounts of *Hsp70 *mRNA were not different between Foundiougne and Missirah. The *Naka *mRNA levels exhibited a pattern similar to *Hsp70 *between locations, being highest at the most saline station, Kaolack, but lowest at the least saline location of Missirah (Figure 1). Where the salinity was intermediate, at Foundiougne, there were intermediate *Naka *mRNA levels.

### Condition factor

**Figure 2:**
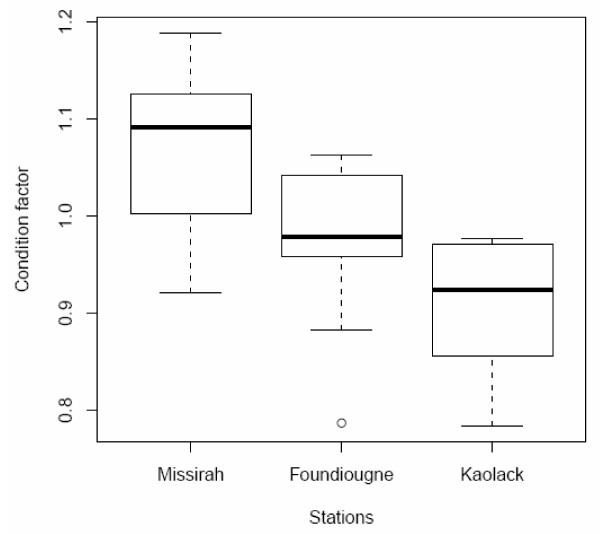
**Condition factor of the black-chinned tilapia *S. melanotheron *from three locations of the Saloum estuary**. Data are illustrated in box plots (in log_2_) that contained the median (horizontal line) as well as the 25th and 75th percentiles (bottom and top edges of the boxes).

The average condition factor (*K*) varied significantly between sites and salinities (Figure 2). Fish caught in Kaolack, the most saline location, had the lowest condition, significantly lower than at Missirah, the least saline location, where the best condition was recorded. Salinity at Foundiougne was intermediate, with no significant difference from the other two sites.

### Correlations between Salinity, mRNA levels and condition factor

**Figure 3:**
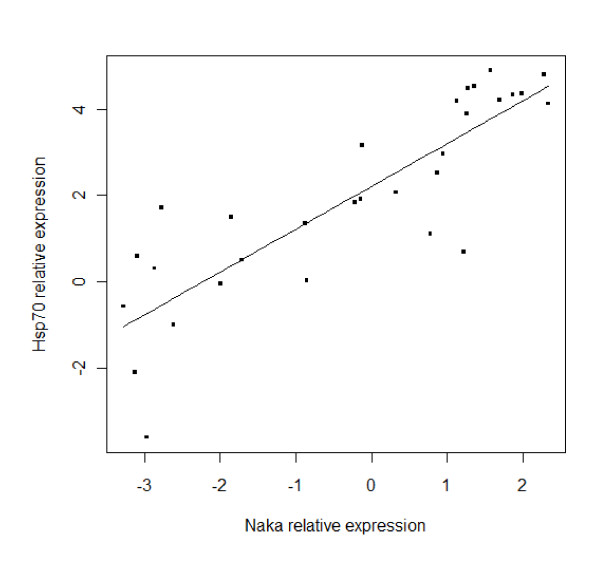
**Relationship between mRNA expression levels of *Hsp70 *and *Naka *(Y = 0.994X + 2.204; R^2 ^= 0.727; *P *< 0.001)**. The mRNA expression levels represent the relative expression normalized to β-actin and are expressed relative expression as log2-transformed data.

**Figure 4:**
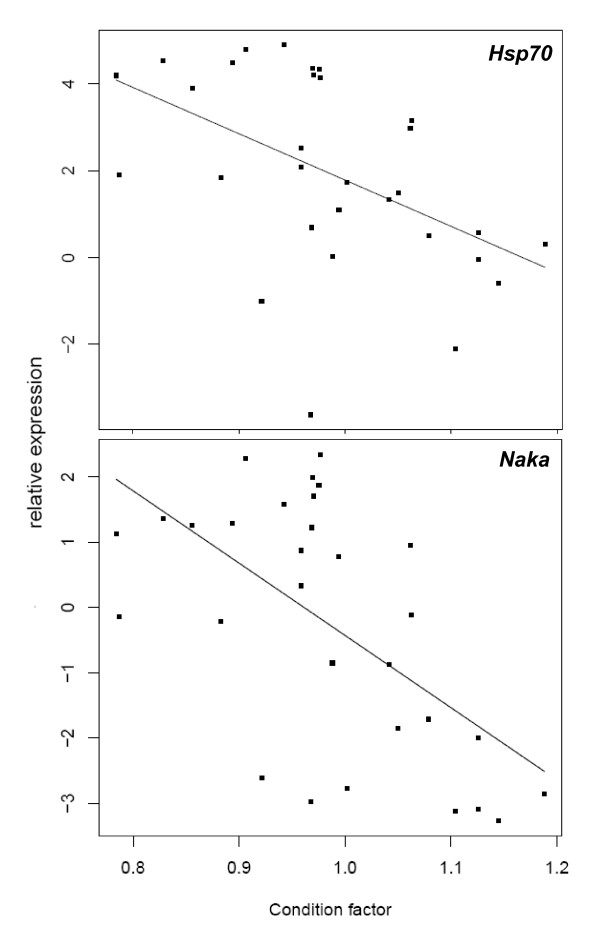
**Relationship between condition factor and mRNA expression levels of *Hsp70 *(Y = -10.665X + 12.447; R^2 ^= 0.241; *P *< 0.001) or *Naka *(Y = -11.053X + 10.623; R^2 ^= 0.352; *P *< 0.001)**. The mRNA expression levels represent the relative expression normalized to β-actin and are expressed relative expression as log2-transformed data.

The results show significant relationships between environmental salinity and the relative expression of *Hsp70 *and *Naka *or condition factor. There was a significant positive correlation between salinity and relative expression of *Hsp70 *(R^2 ^= 0.764; *P *< 0.001) or *Naka *(R^2 ^= 0.855; *P *< 0.001), and a negative correlation of condition factor to salinity (R^2 ^= 0.415; *P *< 0.001). There was a significant positive correlation between *Hsp70 *and *Naka *relative expression (R^2 ^= 0.727; *P *< 0.001) (Figure 3), and a negative relationship between condition factor and relative expression of *Hsp70 *(R^2 ^= 0.241; *P *< 0.001) or *Naka *(R^2 ^= 0.352; *P *< 0.001) (Figure 4).

## Discussion

The Sahelian area of West African is characterized by an extended dry season from November to June, and a short dry season from July to October. In the Senegalese Saloum estuary, salinity levels change between these two seasons [28,32]. The salinity in the estuary decreases during the rainy season due to the input of freshwater by precipitation. In the dry season, however, the salinity increases because of intense evaporation [33]. The fish analysed in this study were collected at the end of the dry season (end of May) when the salinity in the estuary will have been stable for some months. Furthermore, it has been demonstrated that populations of *S. melanotheron *do not undertake large scale movements in the estuaries [34]. Therefore, it is reasonable to consider that fish were acclimatized to the prevailing salinity conditions at the time of collection.

It is, of course, not just salinity that can elicit the induction of stress proteins, but also thermal stressors, oxygen depletion and environmental contaminants [5,35-37]. Previous studies conducted in the Saloum estuary have shown that the water temperature varies only slightly between locations, and that dissolved oxygen is not a limiting factor for the black-chinned tilapia in these areas [29,33,38]. Water turbidity cannot explain overexpression at the hypersaline Kaolack location because it is much more turbid at the estuary mouth than in the upper part of the estuary during the dry season [33]. A heavily polluted location (Hann Bay, 38 psu) [39] did not have higher *Hsp70 *expression levels than at an unpolluted site with similar salinity (Missirah) (see Additional file 1), indicating that differences in pollutant load did not contribute to differential expression. Variations in salinity are therefore the most dominant environmental factor in the Saloum estuary, and salinity is the predominant abiotic stressor.

The present study demonstrates significant correlations between *Hsp70 *and *Naka *expression, environmental salinity and condition factor in natural populations of *S. melanotheron*. The overexpression of *Hsp70 *and *Naka *of the fish living in hypersaline conditions correlates significantly with their lower condition factor.

### Osmoregulatory role of *Hsp70*

If the mRNA levels indicate differences in functionally active proteins, the correlation between *Hsp70 *expression and salinity might reflect a direct role of the stress protein in salinity tolerance by black-chinned tilapia. This is in agreement with the correlations between salinity and the relative expression of *Hsp70 *and *Naka*, and with several *in vitro *studies, where increased NaCl resulted in high *Hsp70 *induction [11,40,41]. Studies recently conducted in the silver sea bream, *Sparus sarba *[12] and the brown trout, *Salmo trutta *[10] have shown concomitant increases in *Hsp70 *and *Naka *mRNA levels in response to hyperosmotic stress. The increase in *Hsp70 *was attributed to a role of this protein in avoiding protein disruption and damage [12]. This function of *Hsp70 *would explain its overexpression in fish living at the extremely hypersaline site (Kaolack, 100 psu). However, our results did not show a significant difference between the fish living in salinity approaching seawater (40 psu) versus relatively hypersaline water (60 psu), suggesting the existence of a threshold for salinity stress and therefore *Hsp70 *overexpression. It has been shown in the black sea bream *S. sarba *that three genes of the *Hsp70 *family (*Hsp70, Hsc70 *and *Hsf1*) were up-regulated in the gills at a salinity of 33 psu, and exhibited an even higher expression in hypersaline water at 55 psu [42]. This was interpreted as a threshold for salt tolerance by the gill in this species which, once exceeded, caused an activation of stress proteins to prevent cell damage. In the black-chinned tilapia, the threshold of salinity tolerance for a significant activation of stress protein appears to be located beyond 60 psu.

### Ecological and evolutionary importance of *Hsp70 *in *S. melanotheron*

Studies in various model organisms have indicated reduced fitness in stressful conditions, which could be directly attributed to the metabolism of stress proteins themselves. That is, the expenditure of more energy on protection by stress protein synthesis would deviate energy from development, growth and reproduction [2]. Consistent with this hypothesis, modified *Drosophila *cells continuallyexpressing *Hsp70 *have reduced growth compared to control cells, but subsequently resume normal growth if the *Hsp70 *is isolated from the cytoplasm [4,5,43]. This may, at least in part, explain why the lowest condition factor occurred in the fish from the most saline location (Kaolack, 100 psu) where the expression levels of *Hsp70 *were highest. Previous studies have shown that the same species from the Kaolack location of the Saloum estuary had reduced growth, poor condition factors, precocious reproduction and lower fecundities compared with the less saline sampling locations [29,30]. The decline in these life history traits in this area could reflect a high energy requirement to meet the increased energetic demands for osmoregulation, in particular the increased expression and activity of ion pumps including the *Naka*. Although it is not simple to establish direct relationships between the *Hsp70 *induction and the fish condition without performing common garden experiments, these impaired growth and poor condition factors could also reflect a high energy requirement for synthesis of proteins needed for the survival at higher salinities [44]. It has also been suggested that, at high concentrations, stress proteins could be toxic and therefore alter or interfere with the normal cellular functions, notably cell growth and development [45]. Therefore, in addition to the energy costs of *Hsp *synthesis, toxic effects of high *Hsp *concentrations could also contribute to differences in growth and condition factors observed between populations of *S. melanotheron *inhabiting the Saloum estuary.

There is, of course, the possibility that differences in growth among populations reflect differences in resources at the sites. In others words, food resources could be more abundant in the environments with lowest salinities. It is also possible that individuals are more effective at foraging when they are not stressed by salinity, and/or that they are more efficient at assimilating nutrients from their prey. These individuals may be able to invest more in growth and reproduction than their counterparts living in hypersaline zones with less energy available for normal biological functions. In this study we do not have measures of food availability, but the opportunistic nature of this species, an omnivore that can exploit many food sources, may indicate that food is not limiting in hypersaline zones of the Saloum estuary.

If the variation of a trait has a genetic basis and affects the fitness of individuals, the variation among populations in *Hsp70 *expression in this study may reflect an action of natural selection. The large spatial and temporal differences of salinity in the environments inhabited by *S. melanotheron*, associated with the central role *Hsp70 *may play in salinity tolerance, makes this gene a potential target for the local selection in this species. The salinity in the Saloum estuary is not only significantly higher at Kaolack location, but the seasonal variations are also larger, with amplitudes that can exceed 70 psu [28]. Interestingly, another member of *Hsps *family, the *Hsc70 *locus has recently been demonstrated to be polymorphic in natural populations of the European flounder, *Platichthys flesus*, inhabiting environments with different salinities [46]. Although this polymorphism has been suggested to arise from the action of natural selection at this locus, the authors were unable to say which of three environmental factors (salinity, temperature or pollution) might be responsible and whether the polymorphism was associated with variation in *Hsc70 *expression and/or activity. In our study, salinity is clearly a potential selective agent which could be responsible for differences in *Hsp70 *expression in *S. melanotheron*, but further analyses of polymorphism in the regulatory regions are required to establish whether there are adaptive polymorphisms at this locus.

## Conclusion

This study provides the first demonstration, in wild fish populations, that *Hsp70 *is involved in long-term acclimatisation to a salinity range between 40 and 100 psu. The data demonstrate that the most hypersaline conditions were stressful for *S. melanotheron*. This chronic stress may be responsible for the impaired growth, precocious reproduction and low fecundity observed at these sites [29,30]. These negative impacts on life history traits may reflect a high energy requirement for osmoregulation and synthesis of proteins needed for the survival at high salinities rather than limited food resources or lower feeding efficiency. The significant correlation of *Hsp70 *mRNA levels with fish condition suggests that this gene may be a potential biomarker of fish health in estuarine environments. Investigating the activity of *Hsp70 *and the mechanisms which regulate its expression in wild population of *S. melanotheron *are interesting topics for future research.

## Methods

### Sampling design

**Figure 5:**
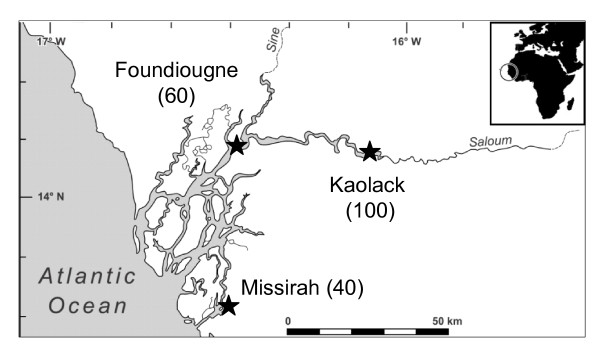
**Sampling locations (black star) of the black-chinned tilapia *Sarotherodon melanotheron *in Saloum estuary (Senegal)**. Fish were collected in May 2006, at the end of the dry season when the most hypersaline conditions were observed in the Saloum estuary. Values in parentheses represent the salinity.

Samples of the black-chinned tilapia *Sarotherodon melanotheron *were collected in May 2006, at the end of the dry season (May) when salinity was relatively stable. Three locations of the Saloum estuary were considered (Figure 5). In this estuary, the locations were respectively Kaolack (the most saline station; 100 psu), Foundiougne (60 psu) and Missirah (40 psu). For each location, the salinity and temperature (Table 1) were measured *in situ *with a refractometer and a thermometer, respectively. Fish sampling was carried out by a local fisherman using castnets with small mesh sizes. To limit fish stress and prevent variability due to manipulation, only five fish were sampled from each castnet thrown. Fish were quickly removed from castnets and anesthetised in 2-phenoxyethanol (2.5 ml l^-1^) before measures of length (fork length, FL, in mm) and mass (total mass, W, in g). Fish were then killed by rapid decapitation and sex as well as gonad maturity stage recorded according to Legendre and Ecoutin [47]. Gills were extracted and stored in *RNA later *(Ambion) at 4°C for 24 h and then at -20°C until processing.

**Table 1:** Sample characteristics of the black-chinned tilapia *Sarotherodon melanotheron *from three wild populations acclimatized to different environmental salinities.

Station	Salinity (psu)	WT (°C)	Sample size	FL range (mm)	W range (g)	Sexe-sexual stage
Missirah	40	28	10	123-160	40.6-84.8	♂-1 (1); ♂-2 (4); ♀-2 (5)
Foundiougne	60	28	10	122-144	39.0-52.2	♂-1 (6); ♀-1 (1); ♀-2 (3)
Kaolack	100	26	10	123-138	32.7-50.6	♂-1 (2); ♂-2 (3); ♀-2 (5)

WT: water temperature; FL: fork length; W: weight. The symbols, (♂, ♀) represent males and females, respectively and the numbers at the side represent the sexual stage. The values in brackets indicate the numbers of individuals analysed for each sex/sexual stage.

Condition factor is a morphometric index frequently used to evaluate physiological status of fish based on the principle that those individuals of a given length which have a higher mass are in better "condition". Assuming that this relationship holds for wild populations, the inter population variation of this index was taken as an indicator of the negative physiological impacts of salinity. The condition factor could be influenced by differences in size or sexual stage. For this reason we performed preliminary analyses which allowed excluding the mature individuals (stages 4 and 5) whose sexual stage seemed to have an influence on the condition factor. Finally, only size classes between 120 and 160 mm fork length with sexual stage 1 or 2, corresponding to immature individuals were analysed (Table 1). Condition factor (*K*) was calculated using the standard formula: *K = 10*^5^*W FL*^-3^; where *W *is the total body mass and *LF *is fork length.

### Total RNA extraction and reverse transcription

Total RNA was extracted from gill tissues stored in *RNA later *(Ambion) using Trizol reagents (Gibco BRL) following the manufacturer's instructions. The RNA concentrations were determined with a spectrophotometer and the RNA integrity was verified by 1% agarose gel electrophoresis. The first strand cDNA was synthesised by reverse transcribing 2 μg total RNA in 20 μL of reaction volume, using MMLV Reverse Transcriptase kit, according to the manufacturer's instructions (Invitrogen).

### Real-time PCR analysis of gene expression

Real-time PCR analysis was used to determine whether changes in selected RNA abundance could be detected from gills sampled from four populations of *S. melanotheron*. Specific primers for the heat shock protein (*Hsp70*) (*Hsp70*F: 5'-ATTGGGTTGCACACCTTCTC-3'; *Hsp70*R: 5'-TGGACAAGTGCAATGAGGTC-3'), Na+, K+-ATPase (*Naka*) (*Naka*F: 5'-ATGAGAAAGCTGAGAGCGAC-3'; *Naka*R: 5'-GGCCTGCATCATACCAATCT-3') and β-actin (β-actinF: 5'-ACAGGTCCTTACGGATGTCG-3'; β-actinR: 5'-CTCTTCCAGCCTTCCTTCCT-3') were designed using Primer 3 software. To determine rt-PCR efficiency of each primer pair used, standard curves were generated using five serial dilutions (1, 1/10, 1/50, 1/100, 1/500) of a unique cDNA sample constituted of a pool of 3 cDNA from each population to be analysed. The rt-PCR quantification was performed on a LightCycler (Roche molecular Biomedicals). Each rt-PCR reaction was conducted in duplicate with an initial denaturation step of 900 s at 95°C followed by an amplification of the target cDNA (40 cycles of denaturation at 95°C for 15 s, annealing between 54°C and 55°C for 15 s, and extension time at 72°C for 15 s). The intra assay variability of rt-PCR was evaluated by calculating the coefficients of variation between duplicates that were all inferior to 10%. Real-time PCR efficiencies (E) were calculated from the given slope of the standard curve according the equation E = 10^(-1/slope)^. The results are presented here as changes in relative expression normalised to the reference gene, β-actin (a gene for which the mRNA abundance in the gills does not change depending on the salinity conditions), using the 2^-(ΔΔCt) ^method described by Pfaffl [48]. β-actin is generally used as a housekeeping gene as well as the 18S RNA, elongation factor 1α (EF 1α) and the GAPDH. We have tested both β-actin and EF 1α and finally chose β-actin as a reference gene because its mRNA levels did not change between our samples.

### Statistical analysis

Condition factor and, *Hsp70 *and *Naka *expression data at each site were expressed as box plots that contained the median as well as the 25th and 75th percentiles. For each of these variables, a Kruskal-Wallis non-parametric test was performed to reveal differences in means between populations. The Mann-Whitney U-test was performed as a post-hoc test. Taking all the individual data from the sites, the strength of the correlations between mRNA levels, environmental salinity and condition factor were assessed by Spearman's rank test. These tests were performed with R or STATISTICA software's. For all tests, a probability of less than 5% (*P *< 0.05) and a confidence of 95% are considered as fiducial level of significance.

## Authors' contributions

JDD, FB and MT conceived the study and participated in its design and coordination. MT performed the lab work, data analyses and manuscript preparation. MT and JDD collected the samples and DM performed the statistical analyses. All the authors contributed equally to the work in discussing research strategy and data interpretation. All authors read and approved the final manuscript.

## Supplementary Material

Additional file 1 **Table showing *Hsp70 *mRNA levels of fish from the Saloum estuary and Hann Bay**. Comparison of *Hsp70 *mRNA levels between fish from Saloum estuary and those collected in a polluted location (Hann Bay). Different superscripts in the last column indicate a significant difference (*P *< 0.05) in *Hsp70 *mRNA levels among locations. Click here for file
