# Supplementary material for: Differential expression of the heat shock protein Hsp70 in natural populations of the tilapia, Sarotherodon melanotheron, acclimatised to a range of environmental salinities
Source: BMC Ecol. 2010 Apr 29;10:11. doi: 10.1186/1472-6785-10-11 (PMC2873927; doi:10.1186/1472-6785-10-11)
Supplement: Additional file 1 — Table showing Hsp70 mRNA levels of fish from the Saloum estuary and Hann Bay. Comparison of Hsp70 mRNA levels between fish from Saloum estuary and those collected in a polluted location (Hann Bay). Different superscripts in the last column indicate a significant difference (P < 0.05) in Hsp70 mRNA levels among locations. [file 1472-6785-10-11-S1.DOC]

| Station | Salinity (psu) | Sample size | Hsp70 relative expression |
| --- | --- | --- | --- |
| Missirah | 40 | 10 | 1.27±1.05bc |
| Foundiougne | 60 | 10 | 4.13±2.63b |
| Kaolack | 100 | 10 | 21.30±4.65a |
| Hann Bay | 38 | 10 | 0.86±0.24c |
